# Development of Pacemaker Lead Thrombosis in a Patient with Atrial Fibrillation during Apixaban Treatment

**Published:** 2019-10

**Authors:** Adem Adar, Orhan Önalan, Fahri Cakan

**Affiliations:** *Department of Cardiology, Faculty of Medicine, Karabuk University, Karabuk, Turkey.*

**Keywords:** *Apixaban*, *Pacemaker, artificial*, *Thrombosis*, *Atrial fibrillation*

## Abstract

Apixaban was introduced in clinical use for nonvalvular atrial fibrillation as an alternative to warfarin. There is a dearth of information regarding apixaban use in patients suffering from atrial fibrillation with intracardiac foreign bodies such as pacemaker leads. In this report, we describe a 72-year-old female patient with a complaint of weakness in both legs of a few days’ duration. She was detected to have a thrombus over the pacemaker lead and inside the left atrial appendage during apixaban treatment. After the discontinuation of apixaban and the commencement of warfarin, the thrombus was resolved. Our case is the first report to show that apixaban treatment (5 mg, twice daily) may not prevent the development of pacemaker lead thrombosis in patients with atrial fibrillation.

## Introduction

Pacemaker lead thrombosis usually develops in conditions predisposing to thrombosis such as heart failure and atrial fibrillation, and its most frightening outcome is pulmonary embolism.^        1 ^ Warfarin has been found to be efficacious in pacemaker lead thrombosis; nonetheless, information regarding the use of apixaban for the treatment and prevention of pacemaker lead thrombosis is lacking. In this report, we present a case with atrial fibrillation and an intracardiac pacemaker lead diagnosed with pacemaker lead thrombosis and left atrial appendage (LAA) thrombosis during apixaban use, which were resolved by apixaban discontinuation and warfarin initiation.

## Case Report

A 72-year-old female patient was admitted to our emergency department with a complaint of weakness in both legs of a few days’ duration. In the initial examination, the patient had stable vital findings and a body temperature of 37.5 ^°^Ϲ with no sign of infection. She had only a history of atrial fibrillation, right heart failure, and intracardiac defibrillator implantation. The pacemaker had been implanted 2 years earlier. It was learned that she had been consuming apixaban (5 mg twice daily), perindopril (10 mg/d), spironolactone (25 mg/d), and furosemide (40 mg/d) for 4 years. Admission blood tests revealed a hemoglobin level of 12.5 g/dL, a white blood cell count of 8390/mm^3^, a platelet count of 238000/mm^3^, a glucose level of 96 mg/dL, a urea level of 20.4 mg/dL, a creatinine level of 0.82 mg/dL, a C-reactive protein level of 10.89 mg/L, and an erythrocyte sedimentation rate of 25 mm/h. 

**Figure 1:**
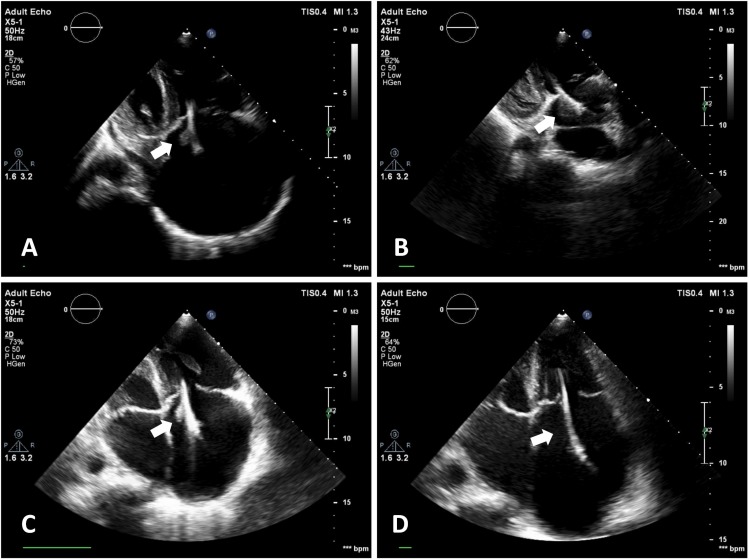
Transthoracic echocardiographic images from the apical 4-chamber view show the stages of the thrombus by time. The thrombus was completely resolved after the initiation of warfarin.

**Figure 2:**
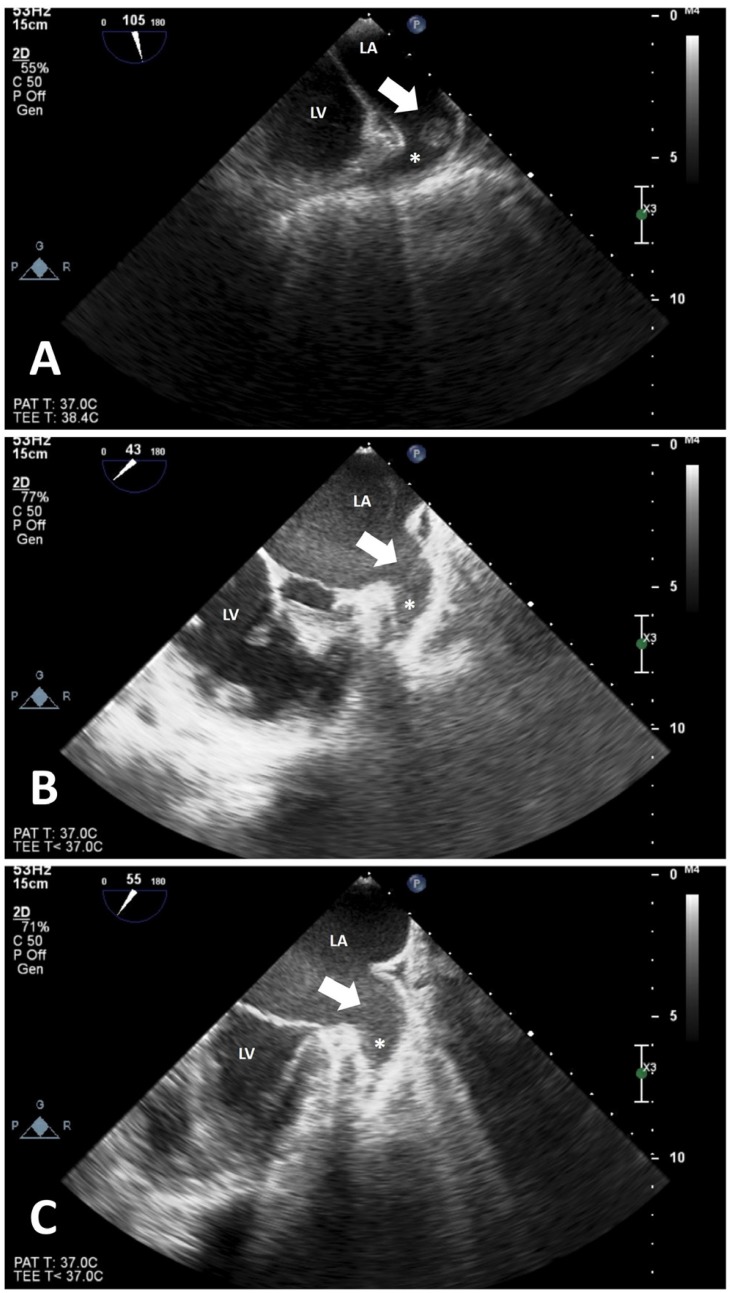
Transesophageal echocardiographic images show the stages of the thrombus by time. The thrombus and the spontaneous echo contrast were completely resolved after the commencement of warfarin.

Electrocardiography demonstrated right bundle branch block and atrial fibrillation. In transthoracic echocardiography (TTE), the ejection fraction was 50%, the right cardiac cavities were dilated, advanced tricuspid insufficiency was present, the pacemaker lead was present in the right cardiac cavities, and a mobile mass image with irregular margins (15×11 mm in size) was observed on the lead (Figure 1A, Video 1). When the clinical condition of the patient was evaluated, the mass image was considered to be possible vegetation; therefore, she was hospitalized with a pre-diagnosis of infective endocarditis. Blood cultures were taken. Head tomography and diffusion magnetic resonance imaging were performed to rule out a stroke. An intravenous empirical therapy with vancomycin and ceftriaxone was initiated for infective endocarditis. Blood culture results were negative. In follow-up TTE a week later, the mass on the lead that had been considered to be vegetation was observed to have enlarged (15×20 mm) (Figure 1B). Thereafter, transesophageal echocardiography (TEE) was performed. In the TEE examination, as a thrombus was observed in the LAA (Figure 2A). The mass image on the lead was considered to be a thrombus. Since apixaban was thought to be possibly inadequate for thrombus resolution, it was discontinued and warfarin was initiated. Antibiotherapy was discontinued on day 7. In follow-up TTE on day 15 of the warfarin treatment, the thrombus was observed to have diminished (10×12 mm) (Figure 1C). In the next follow-up TTE (15 days later), the thrombus on the lead was observed to have been completely resolved (Figure 1D, Video 2). TEE was performed; it demonstrated that the thrombus in the LAA had been partially resolved and there was an intense spontaneous echo contrast (Figure 2B). In the next follow-up TEE (15 days later), the thrombus in the LAA had also been completely resolved (Figure 2C). During the follow-up, the patient did not experience embolic or hemorrhagic events.

A) Thrombus (arrow) is seen on the pacemaker lead in the right atrium.

B) Thrombus (arrow) on the pacemaker lead in the right atrium has enlarged.

C) Thrombus (arrow) on the pacemaker lead in the right atrium has diminished.

D) No evidence of the thrombus is seen on the pacemaker lead in the right atrium (arrow).

LA, Left atrium; LV, Left ventricle; RA, Right atrium; RV, Right ventricle

A) Midesophageal 2-chamber view (80–110°) shows that the thrombus (arrow) in the left atrial appendage.

B) Midesophageal short-axis view (30–60°) shows that the thrombus has been partially resolved and there is also the appearance of an intense spontaneous echo contrast (arrow).

C) Midesophageal short-axis view (30–60°) shows that the thrombus in the left atrial appendage has been completely resolved (arrow).

LA, Left atrium; LV, Left ventricle; ^*^Left atrial appendage

## Discussion

Data regarding the use of novel oral anticoagulation agents in patients suffering from atrial fibrillation with intracardiac foreign bodies are limited. In this report, we presented a case of an atrial fibrillation patient with an intracardiac pacemaker, who was found to have developed pacemaker lead thrombosis and LAA thrombosis during apixaban treatment. 

The pathogenesis of the development of pacemaker lead thrombosis is not fully known; nevertheless, it is known that this condition usually develops in the presence of factors predisposing to thrombus development. In their investigation to detect the risk factors for thrombus formation on pacemaker leads, Rahbar et al.^        1 ^ reported that female sex and the presence of atrial fibrillation could predict the development of lead thrombosis. Therefore, in a similar way, female sex and the presence of atrial fibrillation might have created a predisposition toward the development of lead thrombosis in our patient. Since in our case, we noted 1 major criterion and 1 minor criterion according to the modified Duke criteria, we initiated empirical antibiotherapy, even though the diagnosis of infective endocarditis was not made.^        2^

Patients with pacemaker lead thrombosis may present with a variety of nonspecific symptoms, which may lead to delayed diagnoses. It is, therefore, important that cardiologists pay attention and consider the possibility of pacemaker lead-induced thrombi, especially in high-risk patients and in cases of presumed pacemaker-related infective endocarditis with negative blood cultures. Jesmar Buttigieg et al.^   3 ^ described a middle-aged woman with a vegetation-like structure originating from the pacemaker lead, who underwent open-heart surgery for mass removal. The histology and culture of the retrieved mass confirmed a sterile thrombus with no features to suggest an infected mass. In contrast to that report, we detected a vegetation-like thrombus on the pacemaker lead without an open-heart surgical operation.

Pacemaker leads have high thrombogenicity. An autopsy investigation revealed thrombi in 48% of the atrial leads and 33% of the ventricular leads studied.^   4 ^ In the present case, with atrial fibrillation, the CHADSVasc score was 4. We think that a high cardioembolic risk might have contributed to the development of the lead thrombus. If atrial fibrillation is accompanied by predisposing conditions for the development of thrombi such as the presence of a pacemaker lead, thrombogenicity increases even more, and apixaban might be insufficient for the prevention of thrombus development in this group of patients. We are of the opinion that a marked coagulation activation led not only to the thrombosis on the pacemaker lead but also to the thrombus in the LAA in the present case. Accordingly, we evaluated coagulation disorder parameters such as protein C, protein S, and factor 5 Leiden mutation and the results revealed no coagulation defect. We did not study the level of serum apixaban or anti Xa activity, although we thought that apixaban treatment was inadequate to prevent pacemaker lead thrombosis in patients with atrial fibrillation. On the other hand, guidelines do not recommend investigating this parameter in patients taking new oral anticoagulant drugs.^        5 ^

Although the pharmacokinetic properties of new oral anticoagulant drugs are not predictable, they are thought to be more tolerable and safer than warfarin because of a lack of routine laboratory control and fewer drug interactions. However, it is known that they are not as effective as warfarin in some special cases. Well-known conditions include prosthetic valve diseases and moderate-to-severe rheumatic valve diseases.^   6 ^ Less known conditions are the presence of intracardiac foreign bodies such as pacemaker leads and the presence of left ventricular aneurysms, which increase thrombogenicity. In the present case, our patient, who received apixaban, developed pacemaker thrombosis. Similarly, thrombus development has been reported with the use of other new oral anticoagulants. A previous study reported a pacemaker lead thrombus in a patient with atrial fibrillation treated with rivaroxaban.^   7 ^ In another case, we observed a thrombus in a left ventricular apical aneurysm under dabigatran treatment in a patient with a left ventricular apical aneurysm and atrial fibrillation.^   8 ^ The thrombus was completely resolved after dabigatran was replaced with warfarin.

## Conclusion

Serial echocardiographic follow-ups in case pacemaker lead thrombosis has developed may be beneficial in patients with permanent pacemakers and atrial fibrillation under apixaban treatment.

## Notes:

***This paper should be cited as: ***Adar A, Önalan O, Cakan F. Development of Pacemaker Lead Thrombosis in a Patient with Atrial Fibrillation during Apixaban Treatment. J Teh Univ Heart Ctr 2019;14(4):183-186.

## Notes:

***To watch the following videos, please refer to the relevant URLs: ***

http://jthc.tums.ac.ir/index.php/jthc/article/view/949/872

Video 1. Transthoracic echocardiographic (modified 4-cavity image) shows that the thrombus is on the pacemaker lead in the right atrium.

http://jthc.tums.ac.ir/index.php/jthc/article/view/949/873

Video 2. Transthoracic echocardiographic (modified 4-cavity image) shows that the thrombus on the pacemaker lead in the right atrium has been completely resolved.
